# Efficacy of Spironolactone Treatment in Murine Models of Cutaneous and Visceral Leishmaniasis

**DOI:** 10.3389/fphar.2021.636265

**Published:** 2021-04-13

**Authors:** Valter Viana Andrade-Neto, Juliana da Silva Pacheco, Job Domingos Inácio, Elmo Eduardo Almeida-Amaral, Eduardo Caio Torres-Santos, Edezio Ferreira Cunha-Junior

**Affiliations:** ^1^ Laboratorio de Bioquímica de Tripanosomatídeos, Instituto Oswaldo Cruz, Fundação Oswaldo Cruz, Rio de Janeiro, Brazil; ^2^ Division of Biological Chemistry and Drug Discovery, School of Life Sciences, University of Dundee, Dundee, United Kingdom; ^3^ Laboratório de Imunoparasitologia, Unidade Integrada de Pesquisa em Produtos Bioativos e Biociências, Universidade Federal do Rio de Janeiro, Campus UFRJ-Macaé, Macaé, Brazil

**Keywords:** spironolactone, drug repositioning, leishmania, visceral leishmaniasis, pentavalent antimonial

## Abstract

Translational studies involving the reuse and association of drugs are approaches that can result in higher success rates in the discovery and development of drugs for serious public health problems, including leishmaniasis. If we consider the number of pathogenic species in relation to therapeutic options, this arsenal is still small, and each drug possesses a disadvantage in terms of toxicity, efficacy, price, or treatment regimen. In the search for new drugs, we performed a drug screening of *L. amazonensis* promastigotes and intracellular amastigotes of fifty available drugs belonging to several classes according to their pharmacophoric group. Spironolactone, a potassium-sparing diuretic, proved to be the most promising drug candidate. After demonstrating the *in vitro* antileishmanial activity, we evaluated the efficacy on a murine experimental model with *L. amazonensis* and *L. infantum*. The treatment controlled the cutaneous lesion and reduced the parasite burden of *L. amazonensis* significantly, as effectively as meglumine antimoniate. The treatment of experimental visceral leishmaniasis was effective in reducing the parasite load on the main affected organs (spleen and liver) via high doses of spironolactone. The association between spironolactone and meglumine antimoniate promoted better control of the parasite load in the spleen and liver compared to the group treated with meglumine antimoniate alone. These results reveal a possible benefit of the concomitant use of spironolactone and meglumine antimoniate that should be studied more in depth for the future possibility of repositioning for leishmaniasis co-therapy.

## Introduction

In 2018, among the 200 countries or territories that sent reports to the World Health Organization, 88 were considered endemic for cutaneous leishmaniasis (CL) and 78 were considered endemic for visceral leishmaniasis (VL) (WHO Control of Neglected Tropical Diseases, 2020). It is considered the third most common parasitic disease, after schistosomiasis and malaria, based on morbidity and disability-adjusted life years (DALYs) (GBD 2015 DALYs and HALE Collaborators, 2016). Because of its widespread occurrence, predominantly in the poorest social strata, leishmaniasis is among the most neglected diseases, with very limited investment in diagnosis, treatment, and control (Sunyoto et al., 2019).

Currently, leishmaniasis is treated with a small arsenal of drugs, including pentavalent antimonials, amphotericin B deoxycholate, lipid formulations of amphotericin B, miltefosine, and paromomycin, all of which have disadvantages in terms of toxicity, efficacy, price, or treatment regimen (Agil, 2015). The development of cheaper, safer, and orally available drugs is urgently needed. One way to accelerate this process is the well-known strategy of “old drug, new use research.” Drugs that are currently on the market have already undergone pharmacokinetic and toxicity testing and have been proven to be clinically safe, thus obviating the problematic testing bottleneck of the drug discovery pipeline. One recent example of the successful use of this approach is the demonstration of fexinidazole’s leishmanicidal activity *in vitro* and *in vivo* against *Leishmania infantum*, *L. amazonensis*, and *L. braziliensis* (Wyllie et al., 2012; de Morais-Teixeira et al., 2019).

**FIGURE 1:**
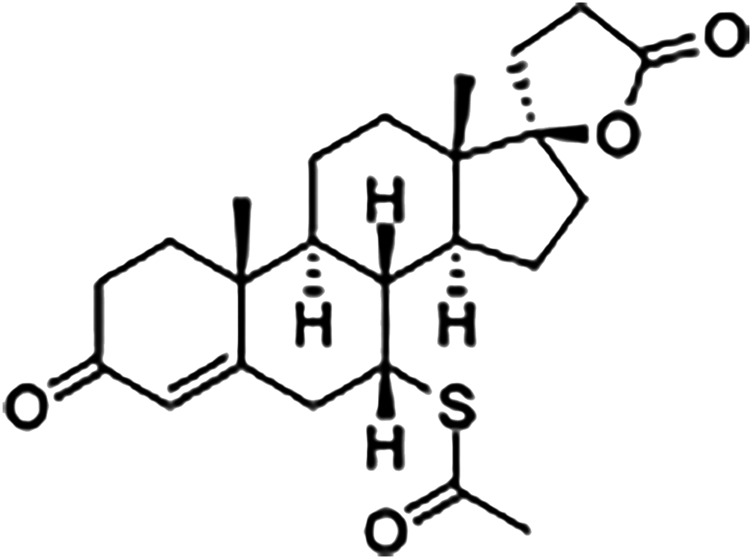
Structure of spironolactone.

Moreover, a recent revision paper published by our group demonstrated that several studies aiming at repurposing drugs for leishmaniasis have been carried out, comprising around 83 drugs (Andrade-Neto et al., 2018). Our group also performed a screening of fifty available drugs belonging to several pharmacological categories; of these, spironolactone (Figure 1), a potassium-sparing diuretic that acts as an aldosterone receptor antagonist, proved to be the most promising drug candidate. Spironolactone was developed in the 1950s, and it is licensed for the treatment of hypertension, heart failure, hypokalemia, and other morbidities (Wile, 2012). In this study, we describe the leishmanicidal activity of spironolactone *in vitro* and its efficacy in treatment alone or in co-therapy with the meglumine antimoniate of experimental cutaneous and visceral leishmaniasis.

## Materials and Methods

### Parasite

Promastigotes of *Leishmania amazonensis* (strain MHOM/BR/77/LTB 0016) and *Leishmania infantum* (strain MHOM/MA/67/ITMAP-263) were maintained at 26°C in Schneider’s insect medium (Sigma–Aldrich, St Louis, MO, United States) with 10% or 20% serum, respectively, with 100 μg/ml streptomycin and 100 U/mL penicillin until the fifth passage. Afterward, old cultures were discarded, and fresh parasites were obtained from infected BALB/c mice.

### *In vitro* Studies

#### Antipromastigote Activity

Promastigotes of *L. amazonensis* and *L. infantum* at 1 × 10^6^ cells/ml were incubated with spironolactone (0–50 μM) or canrenoate (0–128 μM) for 72 h at 26°C. The assays were performed in triplicate in 96-well plates (Costar, New York, United States). Inhibition parasite growth was assessed by a fluorescent assay, Resazurin (Sigma-Aldrich) (Faiões et al., 2018). Briefly, 50 µM of resazurin was added per well, and then the samples were incubated for an additional 2 h. The fluorescence was measured using a Spectra Max GEMINI XPS spectrofluorometer (Molecular Devices, Silicon Valley, United States) at excitation and emission wavelengths of 560 and 590 nm, respectively. The concentration effect curve was fitted for antipromastigote testing via nonlinear regression using Graph Pad Prism 7.0. Finally, the IC_50_ value was determined.

#### Antiamastigote activity

Resident macrophages from BALB/c mice were obtained by peritoneal lavage with 5 ml of cold RPMI medium (Sigma-Aldrich). The cell suspension was adjusted to a 2 × 10^6^/ml concentration and plated in LAB-TEK chambers (Nunc). After 1 h, the cultures were washed with phosphate-buffered saline (PBS) at 37°C to remove non-adherent cells. The remaining cells were incubated at 37°C/5% CO_2_ with promastigotes of *L. amazonensis* at a ratio of 3:1 or *L. infantum* at a ratio of 5:1. After 3 h, the chambers were rewashed to remove free parasites. The monolayers were incubated with spironolactone (0, 2.5, 5.0, and 10.0 µM) or canrenoate (0, 2.0, 4.0, 8.0, and 16 µM) for 72 h at 37°C/5% CO_2_. The antiamastigote activity was evaluated microscopically after staining the chambers with the Instant Prov hematological dye system (Newprov, Curitiba, Brazil); at least 100 macrophages were counted per sample. The results were expressed as the infection index (IF) using the following formula: IF = % infected cells X (number of amastigotes/total macrophages). The concentration effect curve was fitted for the antiamastigote test via nonlinear regression using Graph Pad Prism 7.0, and the IC_50_ value was determined.

#### Cytotoxicity in Murine Macrophages

Mouse peritoneal macrophages in 96-well plates were treated with spironolactone and canrenoate (16–256 μM) for 72 h at 37°C/5% CO_2_. After removing the supernatant, viable cells were quantified by adding resazurin in phosphate buffer saline (PBS), a final concentration of 50 μM, and then the samples were incubated for an additional 2 h. The fluorescence was measured using a Spectra Max GEMINI XPS spectrofluorometer (Molecular Devices, Silicon Valley, United States) at excitation and emission wavelengths of 560 and 590 nm, respectively. The percentage of viable cells was calculated relative to the control cells. The cytotoxic concentrations lethal to 50% of the cells (CC_50_) were obtained by nonlinear regression of the sigmoid growth curves using the Graph Pad Prism 7.0 software.

### Ethics Statement

BALB/c mice were obtained from the Oswaldo Cruz Foundation (FIOCRUZ) animal facilities (Rio de Janeiro, Brazil). The mice were housed in a maximum of four per cage and kept in a conventional room (23 ± 2°C, relative humidity: 60%, with 12 h light-dark cycles). The animals were provided with sterilized water and chow *ad libitum*. Studies in *L. amazonensis* and *L. infantum*-infected BALB/c mice were performed following the guidelines of the Guide for the Care and Use of Laboratory Animals of the Brazilian National Council of Animal Experimentation (COBEA). This study was approved by the Animal Ethics Committee of Oswaldo Cruz Institute (L26/2015).

### *In vivo* Studies

#### Murine model of the Cutaneous Leishmaniasis

BALB/c mice (four per group) were infected in the right ear with 2 × 10^6^ promastigotes of *L. amazonensis* in the stationary phase of growth. Treatment was started seven days after infection. The dose was calculated based on the minimum recommended dose for humans (50 mg/day) and on an adaptation of the formula suggested by Reagan-Shaw et al., as follows: animal dose (mg/kg) = (human Km/animal Km) × human dose (mg/kg), where mouse Km = 3, human Km = 37, and human weight = 70 kg (Reagan-Shaw et al., 2008). The animals were treated orally through an orogastric tube with a suspension of 8.8 mg of spironolactone/kg/day diluted in PBS containing 2% DMSO or were treated intraperitoneally with 30 mg SB^5+^/kg/day of meglumine antimoniate; the control mice were given PBS containing 2% DMSO. All animals were treated five times per week for 6 weeks. The disease’s course was monitored by measuring the thickness of the infected ear with a caliper (Mitutoyo, São Paulo, Brazil) every 3 or 4 days for 49 days.

At the end of the experiment, the animals were euthanized by controlled CO_2_ inhalation. Ears containing the lesions were removed to analyze the parasite load by limiting dilution analysis (LDA), as described previously (da Cunha-Júnior et al., 2011). Tissues were macerated in Schneider medium containing 10% FBS. The cell suspensions were then adjusted to 10 mg tissue/mL in triplicate and were serially diluted 1:2 in 96-well plates, resulting in a final volume of 200 μL/well. The cultures were observed for seven days using an inverted optical microscope to determine the lowest dilution containing parasites. The parasite load was expressed as the number of parasites/mg of tissue. A two-way ANOVA with a Bonferroni post-test was applied in the data analysis.

#### Murine Model of Visceral Leishmaniasis

BALB/c mice were infected intraperitoneally with 1.0 × 10^8^ promastigotes of *L. infantum* stationary phase. Seven days after infection, once the infection had already been established (Cunha-Júnior et al., 2016), treatment with spironolactone was introduced daily in three different doses of 4.4, 8.8, and 17.6 mg/kg/day. Control groups were treated with meglumine antimoniate (28 mg/kg/day of Sb^5+^) intraperitoneally. To determine the association between spironolactone and meglumine antimoniate, a fixed dose of meglumine antimoniate was used (28 mg/kg/day of Sb^5+^). After 23 days of treatment, the animals were euthanatized in a CO_2_ chamber, and the organs of interest (liver and spleen) were aseptically removed, weighed, and homogenized in Schneider’s medium supplemented with 20% HIFCS. The parasitic load was estimated using a parasite LDA. Briefly, the resulting cell suspensions were serially diluted and evaluated by limiting dilution analysis after 7 days (Buffet et al., 1995). The number of parasites/organs was calculated as follows (geometric mean of titer from triplicate cultures) × (common fraction of the homogenized organ added to the first well) × (weight of organ in mg).

## Results

### *In vitro* Activity

The direct antileishmanial activity of spironolactone was first evaluated by incubating *L. amazonensis* or *L. infantum* promastigotes with the drug for 72 h. Spironolactone was a potent inhibitor of parasite growth, with an IC_50_ of 3.6 and 28.6 μM, respectively (Table 1). Next, we evaluated the spironolactone activity against the mammalian evolutive form of the parasite. Spironolactone was found to be active against intracellular amastigotes, with an IC_50_ of 1.8 µM for *L. amazonensis* and 18.0 µM for *L. infantum* (Table 1). The CC_50_ of spironolactone for peritoneal murine macrophages was 150 μM, demonstrating a selectivity index (SI) of 83.3 and 8.3 for *L. amazonensis* and *L. infantum*, respectively (Table 1). Then, we evaluated one of the spironolactone metabolites (potassium canrenoate) against *Leishmania amazonensis*. Interestingly, canrenoate, even at the highest concentration tested (128 μM), was not active against promastigotes but showed an IC_50_ of 7.6 µM in intracellular amastigotes, with less cytotoxicity (>256 µM) in macrophages compared to spironolactone (Table 1).

**TABLE 1:** IC_50_ values for spironolactone and metabolite canrenoate on *L. amazonensis* and *L. infantum* promastigotes and intracellular amastigotes and CC_50_ values for murine peritoneal macrophages and the selectivity index (SI).

	Murine macrophage CC_50_ (µM)	*L. amazonensis*			*L. infantum*		
		Promastigote IC_50_ (µM)	Amastigote IC_50_ (µM)	SI	Promastigote IC_50_ (µM)	Amastigote IC_50_ (µM)	SI
Spironolactone	150.0 ± 3.4	3.6 ± 1.0	1.8 ± 0.7	83.3	28.6 ± 2.2	18.0 ± 1.1	8.3
Canrenoate	>256	>128	7.6 ± 2.3	>34.6	NT	NT	—

Values represent the mean ± SE of three different experiments. NT–not tested.

### *In vivo* Efficacy

**FIGURE 2:**
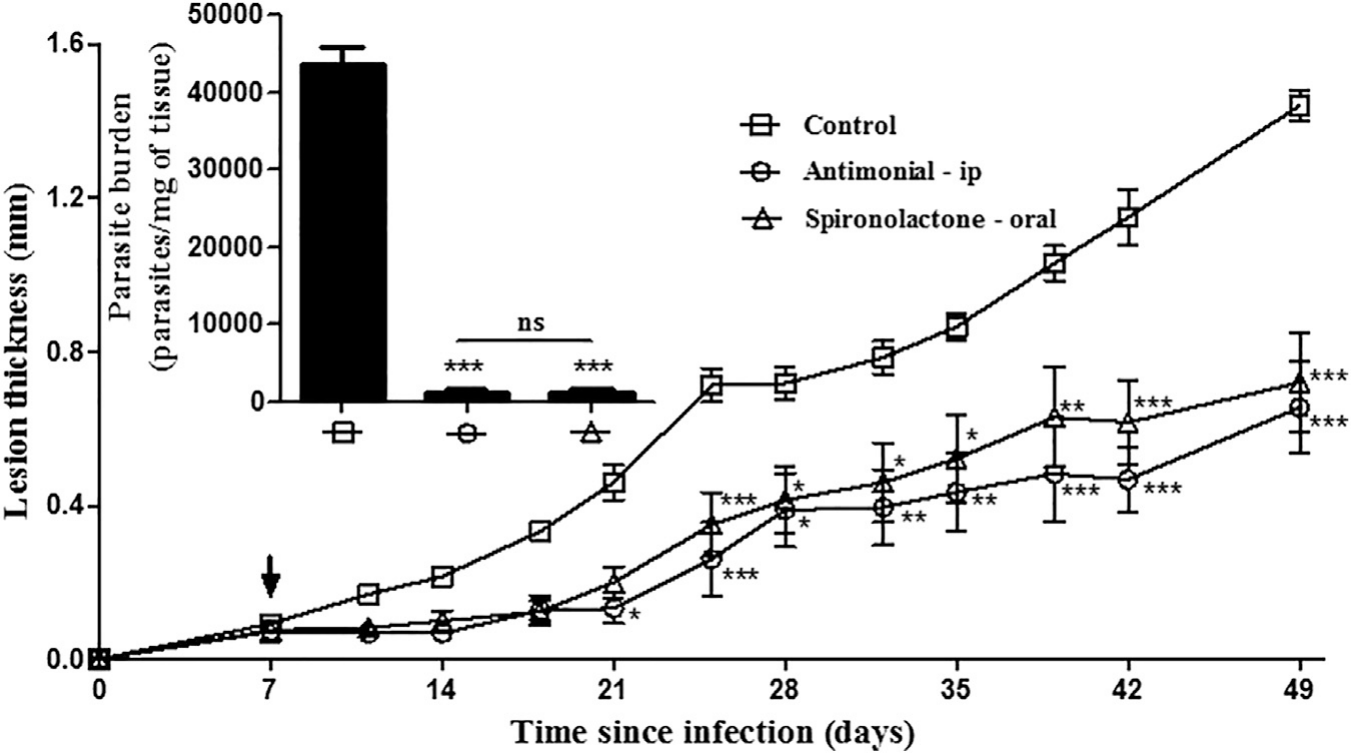
Efficacy of oral spironolactone against murine cutaneous leishmaniasis. BALB/c mice were infected subcutaneously in the right ear with 2 × 10^6^ promastigotes of *L. amazonensis*. All animals were treated five days/week for six weeks. Spironolactone at 8.8 mg/kg/day was diluted in PBS containing 2% DMSO and was given orally; meglumine antimoniate (30 mg/kg/day of the Sb^5+^) or PBS containing 2% DMSO was injected intraperitoneally in the right lower quadrant of the abdomen. The lesion development was monitored two times a week, measuring the infected ear with a caliper. At the end of the treatment, the animals were euthanized, and the parasite load was estimated by limiting dilution analysis (insert). Data are represented as the means ± standard error of four animals per group. *p < 0.05, **p < 0.01, and ***p < 0.001 in treated groups x control group.

After demonstrating the *in vitro* antileishmanial activity, we evaluated the efficacy on a murine experimental model of leishmaniasis using *L. amazonensis* or *L. infantum*. Spironolactone, orally delivered at 8.8 mg/kg five times per week (6 weeks–30 doses), was effective in controlling lesion development from the third week (Figure 2) and the parasite burden (insert Figure 2) in mice. There was no significant difference in either parameter evaluated between the treatment of spironolactone orally and meglumine antimoniate intraperitoneally.

**FIGURE 3:**
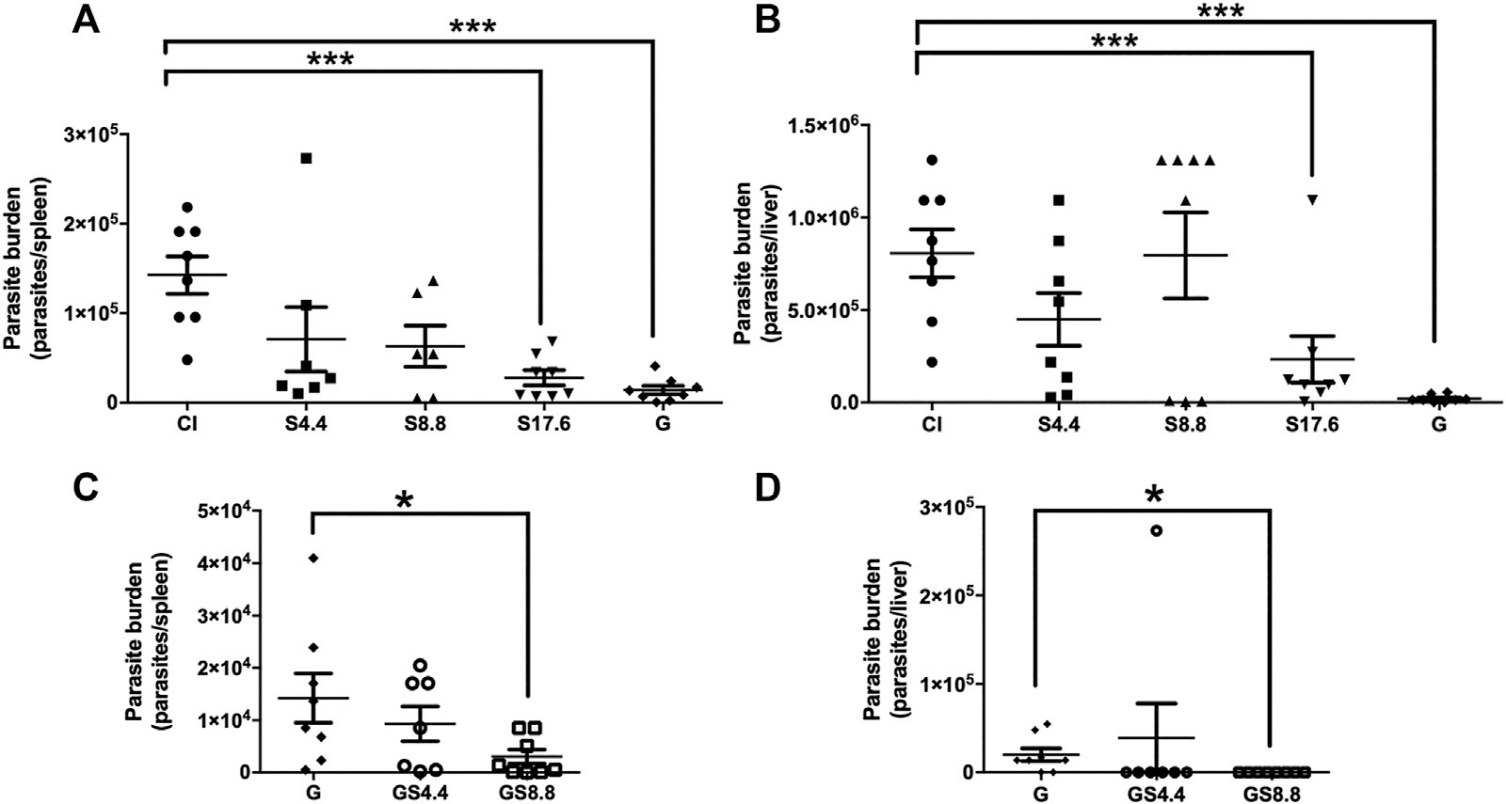
Efficacy of oral spironolactone alone **(A,B)** and its association with meglumine antimoniate **(C,D)** in Experimental Visceral Leishmaniasis. BALB/c mice (8 per group) were infected intraperitoneally with 1.0 × 10^8^ promastigotes of stationary phase *L. infantum*. Seven days after infection, the animals were treated with spironolactone in three different doses of 4.4, 8.8, and 17.6 mg/kg/day along with meglumine antimoniate (28 mg/kg/day of Sb^5+^) by intraperitoneal route. The control group was treated with the vehicle via an oral route (PBS containing 2% DMSO). Regarding the association between spironolactone and meglumine antimoniate, a fixed dose of meglumine antimoniate (28 mg/kg/day of Sb^5+^) was used with 4.4 or 8.8 mg/kg/day of spironolactone. After 23 days of treatment, the animals were euthanized in a CO_2_ chamber, and the spleen (panels **A** and **C**) and liver (panels **B** and **D**) were aseptically removed, weighed, and homogenized. The parasite load was estimated using the limiting dilution assay. Data are presented as means ± standard error of 8 animals per group; *p < 0.05 and ***p < 0.001; CI: control infected; S: spironolactone; and G: meglumine antimoniate.

Experimental visceral leishmaniasis was evaluated in *L. infantum-*infected BALB/c mice. Treatment using 17.6 mg/kg/day of spironolactone was effective in reducing the parasite load on the main affected organs (spleen and liver) (Figures 3A,B). However, it was less effective than the antimonial. To evaluate the benefit of the concomitant use of potassium-sparing diuretic with meglumine antimoniate, we associated the effective dose of meglumine antimoniate with the two lower doses of spironolactone (4.4 mg/kg/day and 8.8 mg/kg/day). The association promoted better control of the parasite load in the spleen compared with the antimonial alone (98 % × 90 % of reduction of the parasite load, respectively). In the liver, we noted a reduction of 97% in the group treated with meglumine antimoniate, while the association reduced 100% of the parasite load (Figures 3C,D).

## Discussion

Drug development is a long and expensive process, taking on average fifteen years of massive investment to generate a product. In leishmaniasis, there was a 60-years gap between the release of pentavalent antimonials and the release of miltefosine (Dorlo et al., 2012; Sundar and Singh, 2016). The development of miltefosine, new formulations of conventional drugs, and combination treatments in recent years have represented a breakthrough for the chemotherapy of leishmaniasis. However, little impact has been observed in endemic areas, which can partly be explained by the high cost of these new alternatives, hindering their use in developing countries (Dorlo et al., 2012). Thus, repositioning drugs seems a suitable tool to search for new therapeutic options for the treatment of neglected diseases.

Here, we present the results of a project that aimed to study the antileishmanial activity of drugs currently used for other pathologies. Spironolactone, the most promising of these drugs, exhibited IC_50_ values of 1.8 and 18.0 µM against the intracellular amastigotes of *L. amazonensis* and *L. infantum*, respectively (Table 1). Approximately 79% of the orally administered dose of spironolactone in humans is converted into canrenone, the most biologically active metabolite described for this drug (Gómez et al., 2005). This metabolite is hydrolyzed in its *γ*-lactone ring, generating canrenoate (soluble in water). After equilibrium, it reaches plasma concentrations like canrenone (Karim, 1978). The reverse also occurs; Karim et al. (1971) demonstrated that after intravenous administration of potassium canrenoate, lactonization of the hydroxy acid group of the canrenoate occurs, producing the *γ*-lactone ring of the canrenone. In 3 h, equal concentrations of these two metabolites have already been found in plasma (Karim et al., 1971). We evaluated the activity of canrenoate and found that this metabolite demonstrates no activity against the promastigotes of *L. amazonensis* but remains active against intracellular amastigotes, although slightly less potent.

After demonstrating the *in vitro* selectivity of spironolactone, its ability to control the development of cutaneous leishmaniasis was evaluated. The infection of BALB/c mice with *L. amazonensis* is well established in the literature as a model of extreme susceptibility, with progressive swelling at the inoculation site accompanied by ulceration and metastasis (Neal and Hale, 1983). Oral treatment with spironolactone effectively controlled the development of the lesion and reduced the parasite load, with activity similar to that of meglumine antimoniate, which was administered intraperitoneally (Figure 2). Spironolactone treatment in the visceral leishmaniasis model showed efficacy, and the combination with meglumine antimoniate was promising. The combination of meglumine antimoniate with 8.8 mg/kg/day of spironolactone was more effective than treatment with meglumine antimoniate alone. Some studies have shown that the use of meglumine antimoniate associated with other drugs is effective; for example, combinations with lapachone derivatives, azoles, amiodarone, and aminosidine sulfate have shown a greater effectiveness when compared to the use of meglumine antimoniate alone (Oliva et al., 1998; Anversa et al., 2017; Gonçalves-Oliveira et al., 2019).

Giannitrapani et al. (2009) described the case of a patient with visceral leishmaniasis who was previously diagnosed with cirrhosis. He was referred on admission for previous treatment with spironolactone and vitamin K. The disease was advanced, and therapy with liposomal amphotericin B was instituted with good response. As it was not a controlled study, no conclusions can be drawn about how spironolactone influenced the development of the disease. In addition, data on dosage and treatment regimen were not mentioned. It is worth mentioning that spironolactone has contraindications for patients with anuria, acute renal failure, significant impairment of renal excretory function, or hyperkalemia. Thus, it should be used with caution in some cases, as described in the product monograph (Pfizer, 2015).

Spironolactone has a nucleus like other spirolactones, which are described as inhibitors of 17β-hydroxysteroid dehydrogenase (17β-HSD), an enzyme involved in the last stage of cholesterol biosynthesis (Poirier et al., 2001). In addition, spironolactone has also been implicated as an inhibitor of CYP90B1, which is responsible for the biosynthesis of brassinosteroid phytohormone in plants (Asami et al., 2004; Bajguz et al., 2020). Thus, a possible mechanism of antileishmania activity of this drug is interference in the sterol biosynthesis of the parasite. This pathway is considered essential since its pharmacological inhibition results in the death of the parasite (Roberts et al., 2003). However, specific experiments to evaluate this hypothesis must be carried out.

In conclusion, these results demonstrate a remarkable effect of spironolactone on murine cutaneous and visceral leishmaniasis. Additional studies are needed to determine the activity of spironolactone against other *Leishmania* species to optimize the therapeutic regimen and to determine its mechanism of action.

## Data Availability

The raw data supporting the conclusion of this article will be made available by the authors, without undue reservation.
